# Supplementary material for: Exploring how people with dementia can be best supported to manage long-term conditions: a qualitative study of stakeholder perspectives
Source: BMJ Open. 2020 Oct 8;10(10):e041873. doi: 10.1136/bmjopen-2020-041873 (PMC7545621; doi:10.1136/bmjopen-2020-041873)
Supplement: Supplementary data [file bmjopen-2020-041873supp001.pdf]

PROCESS OF SUBSTITUTING SELF-MANAGEMENT	Files	References
**Autonomy verses risk**	0	0
Consequences of non-adherence	2	3
Independence	5	8
Lock safe for medication	4	7
Reliance on others	3	6
Risk management	9	11
Safety concerns	8	14
**Stages of dementia**	5	5
Ability to self-manage	9	9
Capacity	5	8
Managing a spectrum of needs	5	6
Support increasing with level of need	7	9
Taking responsibility for tasks	8	11

**Support in medication management**	0	0
Blister packs	12	18
Encouragement to take medication	5	5
Family member managing medication	15	20
Homecarer role in medication management	26	63
Training to improve limits to HCW role	6	9
Memory aids	8	9
Prompts	9	11
Role of HCP in medications	7	8
Understanding medication	13	15

Rees JL, *et al. BMJ Open* 2020; 10:e041873. doi: 10.1136/bmjopen-2020-041873

COMMUNICATION IN THE CARE NETWORK	Files	References
**Appointment**	13	25
Family carer support in medical appointments	14	23
Organising support and care	12	14
Seeking and providing advice	3	4
**Continuity of care**	8	12
Relationships	7	8
Understanding PLWD	8	9
**Monitoring change**	11	12
Medication review	8	9
Review and monitoring	8	8
**PLWD communication**	9	12
Communication difficulties	7	12
Family carers proxy	4	4

Rees JL, *et al. BMJ Open* 2020; 10:e041873. doi: 10.1136/bmjopen-2020-041873

IMPACT ON AND FROM DEMENTIA	Files	References
Aligned goals and different priorities	5	9
**Decision making**	8	9
**Inter-relateness of physical health and cognition**	0	0
Dementia	7	8
Link between cognition and physical health	15	29
Managing dementia when supporting physical health	7	11
Physical health problems resulting in dementia diagnosis	8	12
**Services congruent to need**	1	1
Accessibility	17	27
Dementia services	3	5
HCP frustration with use of services	1	4
