# Supplementary material for: Exploring how people with dementia can be best supported to manage long-term conditions: a qualitative study of stakeholder perspectives
Source: BMJ Open. 2020 Oct 8;10(10):e041873. doi: 10.1136/bmjopen-2020-041873 (PMC7545621; doi:10.1136/bmjopen-2020-041873)
Supplement: Author's manuscript [file bmjopen-2020-041873.draft_revisions.pdf]

BMJ Open is committed to open peer review. As part of this commitment we make the peer review history of every article we publish publicly available.

When an article is published we post the peer reviewers' comments and the authors' responses online. We also post the versions of the paper that were used during peer review. These are the versions that the peer review comments apply to.

The versions of the paper that follow are the versions that were submitted during the peer review process. They are not the versions of record or the final published versions. They should not be cited or distributed as the published version of this manuscript.

BMJ Open is an open access journal and the full, final, typeset and author-corrected version of record of the manuscript is available on our site with no access controls, subscription charges or pay-per-view fees (http://bmjopen.bmj.com).

If you have any questions on BMJ Open's open peer review process please email info.bmjopen@bmj.com

BMJ Open

Exploring how people with dementia can be best supported to manage long-term conditions: A qualitative study of stakeholder perspectives

Journal:	*BMJ Open*
Manuscript ID	bmjopen-2020-041873
Article Type:	Original research
Date Submitted by the Author:	23-Jun-2020

Complete List of Authors:	Rees, Jessica; University College London, Division of Psychiatry<br>Burton, Alexandra; University College London, Division of Psychiatry<br>Walters, Kate; University College London, Primary Care and Population Health<br>Leverton, Monica ; University College London, Division of Psychiatry<br>Rapaport, Penny; University College London, Division of Psychiatry<br>Herat Gunaratne, Ruminda ; University College London, Division of Psychiatry<br>Beresford-Dent, Julie; University of Bradford<br>Cooper, Claudia; University College London

Keywords:	Dementia < NEUROLOGY, PRIMARY CARE, QUALITATIVE RESEARCH

SCHOLARONE™
Manuscripts

I, the Submitting Author has the right to grant and does grant on behalf of all authors of the Work (as defined in the below author licence), an exclusive licence and/or a non-exclusive licence for contributions from authors who are: i) UK Crown employees; ii) where BMJ has agreed a CC-BY licence shall apply, and/or iii) in accordance with the terms applicable for US Federal Government officers or employees acting as part of their official duties; on a worldwide, perpetual, irrevocable, royalty-free basis to BMJ Publishing Group Ltd ("BMJ") its licensees and where the relevant Journal is co-owned by BMJ to the co-owners of the Journal, to publish the Work in this journal and any other BMJ products and to exploit all rights, as set out in our licence.

The Submitting Author accepts and understands that any supply made under these terms is made by BMJ to the Submitting Author unless you are acting as an employee on behalf of your employer or a postgraduate student of an affiliated institution which is paying any applicable article publishing charge ("APC") for Open Access articles. Where the Submitting Author wishes to make the Work available on an Open Access basis (and intends to pay the relevant APC), the terms of reuse of such Open Access shall be governed by a Creative Commons licence – details of these licences and which Creative Commons licence will apply to this Work are set out in our licence referred to above.

Other than as permitted in any relevant BMJ Author's Self Archiving Policies, I confirm this Work has not been accepted for publication elsewhere, is not being considered for publication elsewhere and does not duplicate material already published. I confirm all authors consent to publication of this Work and authorise the granting of this licence.

For peer review only

Exploring how people with dementia can be best supported to manage long-term conditions: A qualitative study of stakeholder perspectives

Authorship:

Jessica Rees[1*], Jessica.rees@ucl.ac.uk

Alexandra Burton[1], a.burton@ucl.ac.uk

Kate Walters[1], k.walters@ucl.ac.uk

Monica Leverton[1], monica.leverton@ucl.ac.uk

Penny Rapaport[1], p.rapaport@ucl.ac.uk

Ruminda Herat-Gunaratne[1], ruminda.hg@gmail.com

Julie Beresford-Dent[2], J.Beresford-Dent@Bradford.ac.uk

and Claudia Cooper[1], claudia.cooper@ucl.ac.uk

[1]University College London, [2]University of Bradford

*Corresponding author: Jessica Rees, UCL Division of Psychiatry, 6th Floor Wing A, Maple House, 149 Tottenham Court Road, London, United Kingdom, W1T 7BN

Key words: Dementia, Long-term Condition, Multi-morbidity, Management, Qualitative, Secondary Analysis, Primary Care.

WORD COUNT: 3990

For peer review only

Abstract

Objectives: To explore how the self-management of comorbid long-term conditions is experienced and negotiated by people with dementia and their carers.

Design: Secondary thematic analysis of 82 semi-structured interviews.

Setting: Community settings across United Kingdom.

Participants: 11 people with dementia, 22 family carers, 19 health professionals and 30 homecare staff.

Results: We identified three overarching themes: 1) *The process of substituting self-management*: Stakeholders balanced the wishes of people with dementia to retain autonomy with the risks of lower adherence to medical treatments. The task of helping a person with dementia to take medication was perceived as intermediate between a personal care and a medical activity; rules about which professionals could perform this activity sometimes felt confusing and impractical; 2) *Communication in the care network:* It was often the family carer who communicated with services, and made decisions about how to implement medical advice. In situations where family carers or homecare workers were not substituting self-management, it could be challenging for general practitioners to identify changes in self-management and decide when to intervene. 3) *Impact of physical health on and from dementia*: Healthcare professionals acknowledged the inter-relatedness of physical health and cognition to adapt care accordingly. Some treatments prescribed for long-term conditions were perceived as unhelpful when not adapted to the context of dementia. Healthcare professionals and homecare workers sometimes felt that family carers were unable to accept that available treatments may not be helpful to people with dementia and that this sometimes led to the continuation of treatments of questionable benefit.

Conclusion: The process of substituting self-management relies on communication in the care network, whilst considering the impact on and from dementia to achieve holistic physical health management. Care decisions must consider people with dementia as a whole, and be based on realistic outcomes and best interests.

Strengths and limitations of the study

- This was a large study involving 82 in-depth interviews with participants.
- Participants were from diverse backgrounds and a range of locations across the UK.
- Interviews included the perspective of homecare staff in addition to people with dementia, family carers and healthcare professionals.
- Secondary analysis was used to explore sections of transcripts related to multi-morbidity in dementia while the original data collection focused on independence in dementia.

For peer review only - http://bmjopen.bmj.com/site/about/guidelines.xhtml

Introduction

Globally, the prevalence of dementia is predicted to rise to 131.5 million by 2050 compared to 46.8 million in 2015 (1). Over 470,000 people on General Practice registers in England have a formal diagnosis of dementia (2). Almost 8 in every 10 people with dementia live with another chronic long-term disease or condition (3). The health needs within this population are complex (4). The most prevalent multi-morbidities: hypertension, diabetes and coronary heart disease (5), can increase the risk of dementia, while conditions such as stroke, epilepsy and depression are also associated with increased dementia risk (3). The optimal management of physical health may help to prevent hospitalisation and slow cognitive decline (6,7).

Self-management is an integral and lifelong task for people with long-term conditions (8). The symptoms of dementia, including loss of memory, disorientation and executive dysfunction frequently reduce an individual's ability to organise and plan self-care (9). The most commonly described impact is the diminishing ability to administer medication safely (10,11). Most people with dementia need help to manage care for comorbid long-term conditions – usually from a family carer or homecare worker, or both (12–14). Partnership between this care network at home and primary care is critical for the management of long-term conditions in dementia (15).

Research has highlighted a need for guidance on how to tailor and individualise care for people with dementia and long-term conditions (16,17). Clinical guidelines aim to promote evidence-based best practice but tend to focus on single conditions, so may not reflect difficulties in managing multi-morbidity in dementia (18–20). Understanding the lived experiences of stakeholders is critical to the development of relevant clinical guidelines, including considering interactions between comorbidities on the ability of an individual to self-manage their care (21). The first step towards service development includes understanding the care needs of people living with dementia and co-existing long-term conditions (4). A recent review of the literature found limited evidence about how self-management, and support by other stakeholders, such as homecare workers, can support long-term condition management in dementia (15).

This paper aims to explore the experiences of people living with dementia, family carers, healthcare professionals and homecare staff, to identify how the management of long-term conditions is best supported in dementia.

For peer review only - http://bmjopen.bmj.com/site/about/guidelines.xhtml

Methods

The NIDUS (New Interventions for Independence in Dementia) study conducted qualitative, semi-structured interviews with people with dementia, family carers, health and social care professionals and homecare staff. The primary analysis of this qualitative data explored how people with dementia can be supported to live as independently as possible in their own homes (22,23). We carried out a secondary analysis of the transcripts to explore the management of long term conditions for people living at home with dementia.

Recruitment

London (Camden and Kings Cross) Research Ethics Services approved the study in November 2017 (reference: 17/LO/1713). People with dementia and family carers were recruited through three UK NHS memory services, private homecare services, an Alzheimer's Society Experts by Experience group and Twitter. Health and social care professionals were recruited through NHS memory services, social services and clinical academics with links to University College London (UCL). For the homecare staff interviews, ten homecare agencies were recruited across England and managers, office support staff and homecare workers were invited to participate. Purposive sampling ensured our sample of people with dementia and family carers was diverse in terms of age, gender, ethnicity, and, for family carers, their relationship to care recipients; and for professionals, their professional roles, and experiences with supporting people with dementia.

Procedure

Participants were invited to take part in semi-structured qualitative interviews between April and September 2018. All participants gave written informed consent. Interviews lasted on average one hour and were conducted in participants' homes, workplaces or at University offices. Interviews followed a semi-structured topic guide which explored how people with dementia live independently at home and what support they need to do so. They were audio-recorded and transcribed verbatim. Across stakeholders, slightly amended versions of the topic guide were used. The interview schedule for people with dementia and family carers included specific questions about how long-term physical or mental health conditions affected the person with dementia's ability to remain living independently at home.

Data analysis

Secondary analysis involves using pre-existing qualitative data collected from previous research studies to investigate new or additional research questions (24). We took an inductive thematic

For peer review only - http://bmjopen.bmj.com/site/about/guidelines.xhtml

approach through focusing on meaning within the data to develop codes (25) using NVivo 12 software (26).

First, the lead author highlighted information in transcripts relating specifically to long-term conditions in addition to dementia. A coding framework was developed with authors (CC, KW, AB) based on line-by-line coding of highlighted sections. The authors systematically labelled codes based on meaningful fragments of transcripts. We met to agree on re-occurring codes, and the lead author applied the coding framework to all transcripts. After completed analysis of all interviews, we reviewed and refined the coding framework by discussing themes corresponding to the research question. We revisited codes looking at commonalities and differences in managing long-term conditions across accounts of all stakeholders for the thematic analysis.

Patient and public involvement

An advisory group of family carers and health professionals provided feedback on the findings from the NIDUS qualitative study which was incorporated into the analysis. We did not directly involve PPI in this secondary analysis, but we have invited members of the advisory group to help us develop our dissemination strategy.

Results

Participant demographics

Qualitative interviews were conducted with 11 people with dementia (Mean Age= 78.6, SD=7.8), 22 family carers (Mean age= 57.7, SD= 14.3), 19 health and social care professionals (Mean Age= 41.4, SD= 10.9) and 30 homecare staff (Mean Age= 48.3, SD=11.6). To respond to the needs and preferences of participants, eight interviews were dyadic involving people with dementia and family carers together. Details of participant characteristics are presented in Tables 1 and 2.

Table 1: Characteristics of participant demographics (person with dementia/family carer)

		Person with dementia	***Family carer***
**Characteristics**		***n***	***n***
Gender	Female	5	12
	Male	6	10
Ethnicity	White British	8	9
	White other	1	0
	Indian	0	6
	Bangladeshi	0	4
	Other	2	3
Living arrangements	Lives alone	5	7
	Lives with relatives	6	15
Type of dementia	Alzheimer's disease	3	8
	Vascular	2	4

For peer review only - http://bmjopen.bmj.com/site/about/guidelines.xhtml

	Other	2	4
	Not specified	4	6

Table 2: Characteristics of participant demographics (professional carers)

		Homecare staff	***Health and social care professional***
**Characteristics**		***n***	***n***
Gender	Female	25	13
	Male	5	6
Ethnicity	White British	22	9
	White other	2	5
	Black/Black British	5	0
	Asian/Asian British	1	0
	Indian	0	2
	Other	0	3
Professional role	Managerial	7	0
	Homecare worker	19	0
	Other Care Staff	4	0
	Commissioner	0	3
	Social worker	0	2
	Dementia lead	0	2
	Service manager	0	2
	Psychologist	0	2
	Support worker	0	2
	GP	0	2
	Geriatrician	0	1
	Nurse	0	1
	Physiotherapist	0	1
	Psychiatrist	0	1

The lead author (JR) extracted information from interview transcripts about long-term conditions discussed across all stakeholders. Interviews with people with dementia (n=9) and family carer (n=22) explicitly spoke about long-term conditions and support needs. Healthcare professionals (n=14) and homecare staff (n=17) included information on specific long-term conditions, but often spoke more generally about medication management and holistic aspects of health for example, falls and mobility. Figure 1 presents a full overview of reported long-term conditions by people with dementia, family carers, health and social care professionals and homecare staff.

Figure 1: Frequency of long-term conditions discussed in interviews

[ADD FIGURE 1]

For peer review only

Findings

We identified three main themes that responded to our research objectives to explore the experiences of people with dementia, family carers, healthcare professionals and homecare staff in managing long-term conditions in dementia. These were: 1) The process of substituting self-management; 2) Communication in the care network; and 3) Impact on and from dementia (when managing physical health).

1) The process of substituting self-management

Autonomy verses risk

People with dementia valued autonomy when managing their physical health. In the earlier stages of dementia, prompting enabled self-management and a sense of 'still carrying on'. The prospect of requiring more help was a concern to this person living with dementia:

> *"You see I'm praying and I'm hoping that I won't need carers because I want to rely on myself. But I know at some stage I won't be able to. I don't know when. Maybe my health will get worse, physically worse, and this dementia will get worse and I will have to."* (Person with Dementia_1, Arthritis and hypertension)

Homecare workers acknowledged the wishes of people with dementia to retain decision-making control where possible and to be involved in their own care:

> *"And when you give tablets, when you don't know and you can't remember, if someone gives you a pile of tablets, I say every time, this is for your memory, this is your thyroid, this is for your Addison's, this is for your blood pressure. And sometimes, you get a bit sad so this is to help you, and I say what the tablets are. And they say, oh thank God you told me because what you're doing is you're controlling them if you don't involve them."* (Homecare Worker_1)

Family carers balanced the wishes of people living with dementia to retain autonomy as far as possible with the potential consequences of lower adherence to medical treatment. Family carers described their discomfort in providing care against the wishes of the person with dementia but acknowledged the importance of such tasks when memory impeded medication administration:

> *"He then lost his eyesight in the left eye, due to the diabetes medication being missed. He was reporting to my mother daily that he is consuming his medication. Of course, because of*

For peer review only - http://bmjopen.bmj.com/site/about/guidelines.xhtml

the dementia, his memory was deceiving him and telling him that he had done it, but this was a past memory" (Family Carer_1, Son)

Prescriptions and role restrictions

Monitoring or administering medication was the most frequently discussed form of support for long-term condition management. While self-administration of medication is a routine part of long-term condition management, when a person with dementia required support of another person, this role occupied an intermediate position between a personal care activity, and a medical task that required a nursing qualification. Rules around who administered medication could be complex and confusing:

> "*Although we were told that the [home]carers are not allowed to medicate, because they're not insured. So, that's a difficult one.*" (Family Carer_2, Son)

One healthcare professional described the difficulties that could arise because of this when prescriptions changed:

> "*It falls through when they're on, say steroids, like emergency steroids if they've got COPD or if they're on antibiotics for infection. Where the person with dementia will get really poorly because they're in boxes and [home]carers can't give out boxes; it has to be in a nomad. So, again, they do get a short straw because it's not their job to be monitoring what's in the box. They just check the nomad.*" (Community mental health nurse)

2) Communication in the care network

In healthcare settings, it was often the family carer who communicated with services, and made decisions about how to implement medical advice. Being an implementer of medical advice but not the doctor, and the recipient of advice but not the patient, sometimes felt like a dilemma for family carers working as 'care partners' for people with dementia:

> "*.... I suppose at the end of the day I should go down and make an appointment to see the GP.... to tell them that I feel she needs that done.... but I don't want to go down and sort of feel that I'm.... telling them what to do, coming heavy-handed on it either. You know you rely on good relations don't you, with the GP and now being the person we dealt with has gone away and knew us and understood us and that. It's like starting all over again.* " (Family Carer_3, Sister)

The above quote highlights the challenges when developed relationships which facilitated communication come to an end.

For peer review only - http://bmjopen.bmj.com/site/about/guidelines.xhtml

Where people with dementia experienced difficulties with communication, family carers offered support in healthcare appointments to ensure correct reporting of symptoms.

> *"…she would tell them things that were factually incorrect and they would believe that they were correct. Because they just thought she might be a bit old and her literacy wasn't great but they assumed what she was saying was correct, and you do. But she was on drugs that were negatively affecting her and things like that…she was on something that were actually negative to her heart."* (Family Carer_4, Son)

Regardless of the presence of a proxy, participants with dementia highlighted the importance of continued acknowledgement within appointments:

> *"It was, I think that's the problem, some people, because you've got diagnosed with it they think, oh he can't digest this, we'll talk to the relative"* (Person with Dementia_2, Visual Impairment)

In situations where family carers or homecare workers were not substituting self-management, it could be challenging for general practitioners to identify changes in self-management and decide when to intervene:

> *"And I think there are a couple of people actually, thinking back, who have been perhaps bordering on the edge, and who were coming in to see me regularly, and then perhaps have stopped, and it's like actually these are the people perhaps who are the most vulnerable. But I'm not quite sure that the mechanisms are very good for picking up on that, you know, the bit where they're in between, where they're potentially sort of just well enough to go out and about, and do things for themselves, but maybe things at home aren't good and they're not coping, and actually, as a GP, maybe it's really difficult to pick up on that."* (General Practitioner_1)

3) Impact on and from dementia (when managing physical health)

Inter-relatedness of cognition and physical health

As the outcomes associated with physical health and cognition were found to be inter-related, healthcare professionals sought to optimise physical and preventative healthcare in ways that improved cognition.

> *"Yes, so, I think, you know, the physical health of someone has a really big impact on how they're dementia... they experience their dementia. So, they might just have a really poor*

For peer review only - http://bmjopen.bmj.com/site/about/guidelines.xhtml

diet which means that... or they might not be able to manage their diabetes and therefore they're feeling rubbish all the time." (Memory Service Manager)

"*Physical problems as well. You know? Because they have, not only possible dementia, but they also may have physical illnesses. Parkinson's, stroke, any sort of medical condition, as well, on top. So, we don't just concentrate on the dementia. We concentrate on the person. And it's a very much a holistic approach."* (Occupational therapist)

Limitations of physical health treatments in people with dementia

Some treatments prescribed for long-term conditions were considered to be unhelpful for people with dementia, for example because the strategies they used depended on memory, and could not or had not been adapted to the context of dementia. In this next quote, a family carer explains how a person with dementia was too impaired to benefit from suggested strategies:

"*Well, she did have a visit from an occupational therapist [following a stroke]. They arranged that for a few weeks but he, it was when she was at her most florid time and he was recommending that she did things like writing a list down of things that interested her and what she would like to do and things like that. But she wouldn't do any of those. And he said, you know, he came about four or five times, no point in me coming because she doesn't take any notice of what I say, or do anything.*" (Family Carer_3, Sister)

Healthcare professionals and homecare workers sometimes felt that family carers were unable to accept that available treatments may not be helpful to people with dementia. They reported that this sometimes led to the continuation of treatments of questionable benefit, even when it went against the preferences of the person with dementia receiving them:

"*I suppose family members are sometimes trying to keep people as they would see them, well and physically well for as long as possible and doing these things that their diabetes or anything else, and so they'll want them to do certain things whereas that's no longer what is important to that person.*" (General Practitioner_2)

"*I have a lady that's quite old and her mother, she wants the physio in and it's just not possible. She physically can't do it, but she thinks it'll keep her strong a little bit. And it's really not doing her any good, even the physio said that. But the daughter wants it.*" (Homecare Worker_2)

The above quotes highlight how the voice of people living with dementia can be lost when determining the benefits of treatment. Family carers appear to adopt an 'all or nothing' approach to

For peer review only - http://bmjopen.bmj.com/site/about/guidelines.xhtml

physical health decision-making due to the lack of flexible interventions which successfully account for dementia.

At other times, family carers and professionals discussed and agreed approaches in collaboration with people with dementia. One family carer described how this was helpful where difficult, end of life decisions, balancing comfort with quality of life needed to be taken:

> *"His kidney, he's got CKD, which is chronic kidney disease, stage four or five, which is the last stage. Kidney is only about 6% working. So because of that, you know... And we've decided not to go on dialysis. Not just we, but the specialist kidney doctor, we went to see him a couple of months ago and he suggested as well not to put him on dialysis because of his age…And he didn't think it was going to be beneficial to him because, you know, even dialysis can take a lot away from you, three hours of being on a machine three times a week. And because also he's got a slightly weak heart, it's probably not good for him to go on dialysis."* (Family Carer_5, Daughter)

Discussion

Main findings

We describe three themes regarding the experiences of people with dementia, family carers, healthcare professionals and homecare staff in managing long-term conditions in dementia. The process of substituting self-management evolved according to advancement of dementia symptoms. Carers acknowledged the wishes of people with dementia to retain a role in decision-making as long as possible, whilst balancing the risk of lower adherence to treatment. Deciding who is most qualified to administer medication was sometimes confusing as the role occupied a position between personal care and medical tasks. When dementia symptoms resulted in communication difficulties, family carers and people with dementia attended healthcare appointments together becoming a dyad in reporting symptoms and implementing advice. People with dementia still expect to be regarded as equal when accompanied by a carer. Changes in an individual's ability to self-manage may be difficult to detect, especially when people with dementia infrequently accessed primary care. A holistic approach to care involved healthcare professionals recognising the inter-relatedness of cognition and physical health and adapting physical healthcare to the context of dementia. There appeared to be a lack of clarity around decision-making at times, which led to the continuation of treatment with questionable benefit, even when it went against the preferences of the person with dementia receiving them and the judgement of professionals. Family carers found it difficult to accept the limitation of treatments resulting in a tension between their beliefs about benefits and the voice of the person with dementia. Increased awareness about likely harm and

For peer review only - http://bmjopen.bmj.com/site/about/guidelines.xhtml

benefit of treatment based on evidence regarding age and stage of dementia is likely to improve best interest decision-making, for example during the difficult process of deciding of ceiling of care to successfully balance comfort with quality of life.

Addressing the research gap recently identified in the field of dementia and multi-morbidity, these findings describe the interacting effects between cognition and physical health and provide an understanding of the care needs of this population (4,21). In dementia care, family carers often experienced a role transition from 'care-partner' to 'care-giver' with increasing disability (12). Our analysis demonstrates the critical role of family carers in communicating with primary care (13). In addition to impacting memory, judgment and orientation, the impact of dementia on language skills has previously been identified as a barrier to reporting symptoms in healthcare appointments (9).

One of the main self-management tasks across long-term conditions is medication adherence (8,9). Similar to the findings of Rapaport et al (2020), our analysis highlights the role of homecare workers in adapting a facilitative approach; specifically 'doing with not for' during medication management. The need for both family carers and homecare workers to prevent harm often conflicts with the desire of a person with dementia to remain autonomous (27). The balancing of roles is complex for carers especially involving boundaries between advocating for independence and the implementation of medical advice (28). Our findings demonstrate this dilemma extends to the management of long-term conditions in dementia.

The integrated logic of care model suggests that psychosocial and physical needs have a great influence on each other, and should therefore be addressed simultaneously (29). Our analysis highlights a similar need for physical health and cognition. One of the five core self-management skills is decision-making (8) impacted by dementia due to impairments in executive functioning (9). In this context, care decisions involving all stakeholders require a focus on quality of life over a target-driven approach (16) which is continuously re-assessed according to changes in disease state (17).

Clinical implications

These findings highlight that management of long-term conditions in dementia exists on a continuum with support from all stakeholders developing across stages of dementia and severity of long-term conditions. To best support people with dementia to manage long-term conditions, treatments should be congruent to need and consider the impact of dementia on engagement. The development of dementia specific consultation models must consider the impact of dementia on language skills and communication ability, and work with family carers acting as proxies or 'care partners.' Greater considerations of how healthcare professionals communicate the limitations of

For peer review only - http://bmjopen.bmj.com/site/about/guidelines.xhtml

treatments that are not in the best interest or of questionable benefit to people with dementia and family carers would improve physical health decision-making. Such discussions are often complex, involving various stakeholders and ensuring the voice of people with dementia is heard when deciding on treatment benefits can be challenging. Service development must identify mechanisms for detecting change in self-management ability when family carer or homecare workers are not involved in supporting self-management at home for example, alerts for appointment non-attendance and medication under-utilisation.

Strengths and limitations

To our knowledge, this paper presents the first qualitative analysis to consider how people with dementia can be best supported to manage long-term conditions from the perspective of all stakeholders. Specifically, this paper expands on previous research by considering the role of the homecare worker in the care network, previously described in the literature as a triad between the person with dementia, their family carers, and healthcare professionals (30,31). The analysis includes interviews from a large and varied sample from a range of locations across the UK.

We used secondary analysis to explore sections of transcripts that discuss multi-morbidity in dementia. A limitation of this analysis approach is that the data was originally collected to explore how to support independence at home in dementia. Nevertheless, participants spontaneously discussed experiences of long-term conditions due to their salience, enabling further exploration of multi-morbidity in this analysis. All family carers explicitly discussed the management of long-term conditions which may account for large representation from this stakeholder in the analysis, while people with dementia, healthcare professionals and homecare workers would discuss more generally about medication management and physical health.

We included co-authors involved in original data collection (AB, ML, JBD, RHG) to bring an inside knowledge of context during analysis to overcome the problem of the lead author 'not having been there' (24). In addition, the lead author listened to recordings of transcripts to become familiar with data. The use of additional methods such as participant observations in future research would enable triangulation of results on how stakeholders can best support people with dementia to manage long-term conditions (32).

Conclusion

Family carers and homecare workers support the management of long-term conditions in the home. Family carers act as proxies for communication in healthcare appointments. Healthcare professionals have a responsibility to identify changes in self-management ability as dementia progresses; and adapt care accordingly. This process of substituting self-management relies on

For peer review only - http://bmjopen.bmj.com/site/about/guidelines.xhtml

communication in the care network, whilst considering the impact on and from dementia to achieve holistic physical health management. Care decisions must consider people with dementia as a whole, and be based on realistic outcomes and best interests.

Declarations

Declaration of interests

The authors declare that they have no known competing financial interests or personal relationships that could have appeared to influence the work reported in this paper.

Funding statement

This work was supported by the Economic and Social Research Council [grant number ES/P0 0 0592/1]; and the National Institute of Health Research (NIHR) Collaboration for Leadership and Applied Health Research (North Thames). The views expressed are those of the author(s) and not necessarily those of the NIHR or the Department of Health and Social Care.

Data collection was carried out within the UCL Alzheimer's Society Centre of Excellence for Independence at home, NIDUS (New Interventions in Dementia Study) programme (Alzheimer's Society Centre of Excellence grant 330).

Author contribution

All authors made a substantial contribution to this work. JR, CC, KW and AB all contributed to the conception and design of the study and JR drafted the paper. All authors critically revised it and gave final approval for this version to be published. AB, ML, JBD, PR and RHG collected the qualitative data. JR, CC, KW, and AB all coded some of the interview transcripts. JR then organised the data into preliminary themes and led the subsequent analysis, CC, KW and AB further refined the thematic analysis in an iterative process.

Data sharing statement

The qualitative data used and analysed during the current study are available from the corresponding author on reasonable request.

References

1. Prince M, Wimo A, Guerchet M, Ali GC, Wu YT, Prina M. World Alzheimer Report 2015. London, UK. Alzheimer's Dis Int [Internet]. 2015;1–92. Available from: https://www.alz.co.uk/research/WorldAlzheimerReport2015.pdf
2. NHS Digital. Recorded Dementia Diagnoses - March 2020 [Internet]. 2020 [cited 2020 May 1]. Available from: https://digital.nhs.uk/data-and-information/publications/statistical/recorded-dementia-diagnoses/march-2020

For peer review only - http://bmjopen.bmj.com/site/about/guidelines.xhtml

3. Public Health England. Dementia: comorbidities in patients - GOV.UK [Internet]. 2019 [cited 2020 Apr 27]. Available from: https://www.gov.uk/government/publications/dementia-comorbidities-in-patients

4. Welsh. Multimorbidity in people living with dementia. Case Reports Women's Heal [Internet]. 2019;23:e00125. Available from: https://doi.org/10.1016/j.crwh.2019.e00125

5. Browne J, Edwards DA, Rhodes KM, Brimicombe DJ, Payne RA. Association of comorbidity and health service usage among patients with dementia in the UK: A population-based study. BMJ Open. 2017;7(3):1–8.

6. Bordier L, Doucet J, Boudet J, Bauduceau B. Update on cognitive decline and dementia in elderly patients with diabetes. Diabetes Metab [Internet]. 2014 Nov 1 [cited 2019 Sep 20];40(5):331–7. Available from: https://www.sciencedirect.com/science/article/pii/S1262363614000366?via%3Dihub

7. Doraiswamy PM, Leon J, Cummings JL, Marin D, Neumann PJ. Prevalence and Impact of Medical Comorbidity in Alzheimer's Disease. Journals Gerontol Ser A Biol Sci Med Sci [Internet]. 2002 Mar 1 [cited 2019 Aug 23];57(3):M173–7. Available from: https://academic.oup.com/biomedgerontology/article-lookup/doi/10.1093/gerona/57.3.M173

8. Lorig KR, Holman HR. Self-management education: History, definition, outcomes, and mechanisms. Ann Behav Med [Internet]. 2003 Aug [cited 2019 Jun 10];26(1):1–7. Available from: http://www.ncbi.nlm.nih.gov/pubmed/12867348

9. Ibrahim J., Anderson. L., Davis., M C, MacPhail. A, Lovell. J, Winbolt. M. Chronic disease self-management support for persons with dementia, in a clinical setting. J Multidiscip Healthc [Internet]. 2017;10:49–58. Available from: http://europepmc.org/search?query=(DOI:%2210.2147/JMDH.S121626%22)%0Ahttp://www.ncbi.nlm.nih.gov/pmc/articles/PMC5283068/

10. Elliott RA, Goeman D, Beanland C, Koch S. Ability of Older People with Dementia or Cognitive Impairment to Manage Medicine Regimens: A Narrative Review. Curr Clin Pharmacol [Internet]. 2015 [cited 2019 Aug 23];10:213–21. Available from: https://www.ncbi.nlm.nih.gov/pmc/articles/PMC5396255/pdf/CCP-10-213.pdf

11. Kaasalainen S, Dolovich L, Papaioannou A, Holbrook A, Lau E, Ploeg J, et al. The process of medication management for older adults with dementia. J Nurs Healthc Chronic Illn [Internet]. 2011 Dec 1 [cited 2019 Aug 23];3(4):407–18. Available from: http://doi.wiley.com/10.1111/j.1752-9824.2011.01114.x

12. Lazaroff A, Morishita L, Schoephoerster G, McCarthy T. Using dementia as the organizing principle when caring for patients with dementia and comorbidities. Minn Med. 2013 Jan;96(1):41–6.

13. Health Education England. Managing success in dementia care [Internet]. 2018. Available from: https://www.hee.nhs.uk/sites/default/files/documents/Managing Success v3.pdf

14. Allen S. Better domiciliary care for people with dementia Preface [Internet]. 2014 [cited 2020 Apr 27]. Available from: www.skillsforcare.org.uk

15. Rees J, Tuijt R, Burton A, Walters K, Cooper C. Supporting self-care of long-term conditions in dementia: A systematic review. Int J Nurs Stud [Internet]. 2020 Sep 25 [cited 2019 Oct 17];103432. Available from: https://www.sciencedirect.com/science/article/pii/S0020748919302391

For peer review only

For peer review only - http://bmjopen.bmj.com/site/about/guidelines.xhtml

16. Bunn F, Goodman C, Jones PR, Russell B, Trivedi D, Sinclair A, et al. Managing diabetes in people with dementia: A realist review. Health Technol Assess (Rockv). 2017;21(75):1–139.

17. Baird C, Woolford MH, Young C, Winbolt M, Ibrahim J. Chronic disease management and dementia: a qualitative study of knowledge and needs of staff. Aust J Prim Health [Internet]. 2019 [cited 2019 Sep 5]; Available from: http://www.publish.csiro.au/?paper=PY18197

18. Guthrie B, Payne K, Alderson P, McMurdo MET, Mercer SW. Adapting clinical guidelines to take account of multimorbidity. BMJ. 2012;345(7878):1–5.

19. Mutasingwa DR, Ge H, Upshur REG. How applicable are clinical practice guidelines to elderly patients with comorbidities? Vol. 57, Canadian Family Physician. 2011.

20. NICE. Multimorbidity: clinical assessment and management. 2016;

21. Subramaniam H. Co-morbidities in dementia: Time to focus more on assessing and managing co-morbidities. Age Ageing. 2019;48(3):314–5.

22. Herat-Gunaratne R, Cooper C, Mukadam N, Rapaport P, Leverton M, Higgs P, et al. ": Caring Experiences of UK Bangladeshi and Indian Family Carers of People Living With Dementia at Home. Gerontol cite as Gerontol [Internet]. 2020 [cited 2020 Mar 5];60(2):331–9. Available from: https://academic.oup.com/gerontologist/article-abstract/60/2/331/5581953

23. Rapaport P, Burton A, Leverton M, Herat-Gunaratne R, Beresford-Dent J, Lord K, et al. "I just keep thinking that I don't want to rely on people." a qualitative study of how people living with dementia achieve and maintain independence at home: stakeholder perspectives. BMC Geriatr. 2020 Jan 3;20(1):5.

24. Heaton J. Secondary analysis of qualitative data: an overview. Hist Soc Res [Internet]. 2008 [cited 2020 May 1];33(3):33–45. Available from: https://doi.org/10.12759/hsr.33.2008.3.33-45

25. Braun V, Clarke V. Using thematic analysis in psychology. Qual Res Psychol [Internet]. 2006 [cited 2019 Aug 28];3(2):77–101. Available from: https://www.tandfonline.com/action/journalInformation?journalCode=uqrp20

26. QSR International Pty. NVivo qualitative data analysis software. 2018. p. Version 12.

27. Smebye KL, Kirkevold M, Engedal K. Ethical dilemmas concerning autonomy when persons with dementia wish to live at home: A qualitative, hermeneutic study Healthcare needs and demand. BMC Health Serv Res [Internet]. 2016 Jan 19 [cited 2020 Apr 28];16(1):21. Available from: http://bmchealthservres.biomedcentral.com/articles/10.1186/s12913-015-1217-1

28. Leverton M, Burton A, Rees J, Rapaport P, Manthorpe J, Downs M, et al. A systematic review of observational studies of adult home care. Health Soc Care Community [Internet]. 2019 Nov 22 [cited 2019 Oct 17];27(6):1388–400. Available from: https://onlinelibrary.wiley.com/doi/abs/10.1111/hsc.12831

29. Hansen A, Hauge S, Bergland Å. Meeting psychosocial needs for persons with dementia in home care services - A qualitative study of different perceptions and practices among health care providers. BMC Geriatr. 2017;17(1):1–10.

30. Fortinsky RH. Health care triads and dementia care: Integrative framework and future directions. In: Aging and Mental Health. 2001.

31. Bunn F, Burn A-M, Goodman C, Robinson L, Rait G, Norton S, et al. Comorbidity and dementia: a mixed-method study on improving health care for people with dementia (CoDem). Heal Serv Deliv Res. 2016;4(8):1–156.

For peer review only - http://bmjopen.bmj.com/site/about/guidelines.xhtml

32. Mays N, Pope C. Qualitative research in health care Assessing quality in qualitative research [Internet]. Vol. 320, BMJ VOLUME. 2000 [cited 2020 Apr 28]. Available from: www.bmj.com

For peer review only

For peer review only - http://bmjopen.bmj.com/site/about/guidelines.xhtml

Figure 1: Frequency of long-term conditions discussed in interviews

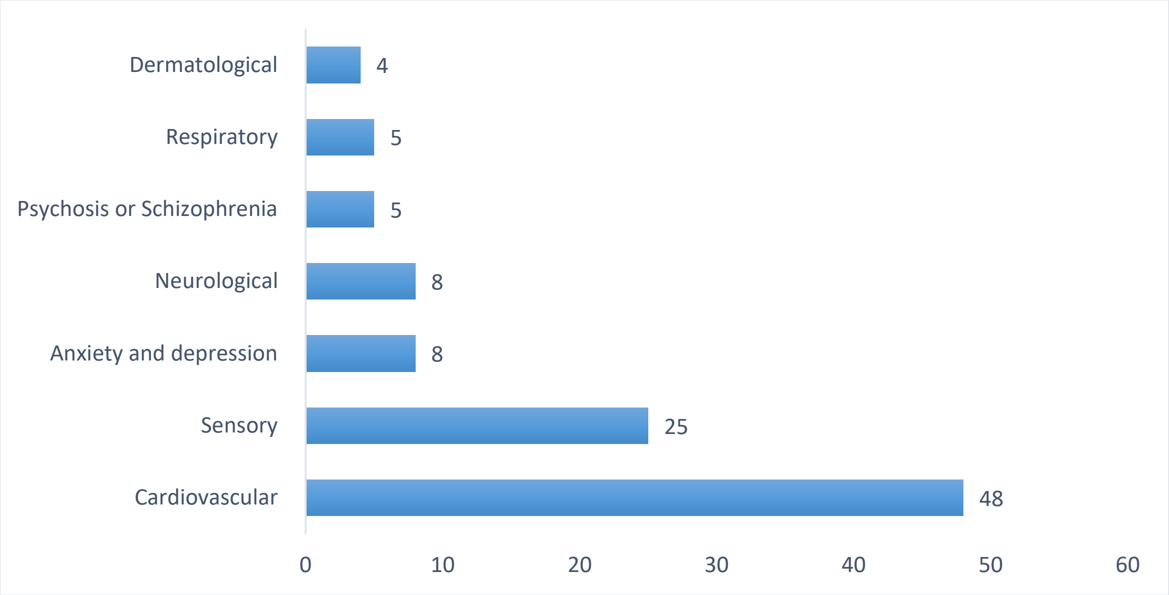

NB:

Category	Long-term condition
**Mental health**	Anxiety and depression<br>Psychosis and Schizophrenia
**Sensory**	Visual impairment<br>Hearing impairment
**Musculoskeletal**	Arthritis<br>Osteoporosis
**Respiratory**	Asthma<br>Pulmonary fibrosis<br>Chronic obstructive pulmonary disease
**Cardiovascular**	Diabetes<br>Hypertension<br>Heart problems<br>Stroke
**Dermatological**	Eczema<br>Psoriasis<br>Dermatitis

COREQ (COnsolidated criteria for REporting Qualitative research) Checklist

A checklist of items that should be included in reports of qualitative research. You must report the page number in your manuscript where you consider each of the items listed in this checklist. If you have not included this information, either revise your manuscript accordingly before submitting or note N/A.

Topic	Item No.	Guide Questions/Description	Reported on Page No.
**Domain 1: Research team and reflexivity**			
Personal characteristics			
Interviewer/facilitator	1	Which author/s conducted the interview or focus group?	
Credentials	2	What were the researcher's credentials? E.g. PhD, MD	
Occupation	3	What was their occupation at the time of the study?	
Gender	4	Was the researcher male or female?	
Experience and training	5	What experience or training did the researcher have?	
Relationship with participants			
Relationship established	6	Was a relationship established prior to study commencement?	
Participant knowledge of the interviewer	7	What did the participants know about the researcher? e.g. personal goals, reasons for doing the research	
Interviewer characteristics	8	What characteristics were reported about the inter viewer/facilitator? e.g. Bias, assumptions, reasons and interests in the research topic	
**Domain 2: Study design**			
Theoretical framework			
Methodological orientation and Theory	9	What methodological orientation was stated to underpin the study? e.g. grounded theory, discourse analysis, ethnography, phenomenology, content analysis	
Participant selection			
Sampling	10	How were participants selected? e.g. purposive, convenience, consecutive, snowball	
Method of approach	11	How were participants approached? e.g. face-to-face, telephone, mail, email	
Sample size	12	How many participants were in the study?	
Non-participation	13	How many people refused to participate or dropped out? Reasons?	
Setting			
Setting of data collection	14	Where was the data collected? e.g. home, clinic, workplace	
Presence of non-participants	15	Was anyone else present besides the participants and researchers?	
Description of sample	16	What are the important characteristics of the sample? e.g. demographic data, date	
Data collection			
Interview guide	17	Were questions, prompts, guides provided by the authors? Was it pilot tested?	
Repeat interviews	18	Were repeat inter views carried out? If yes, how many?	
Audio/visual recording	19	Did the research use audio or visual recording to collect the data?	
Field notes	20	Were field notes made during and/or after the inter view or focus group?	
Duration	21	What was the duration of the inter views or focus group?	
Data saturation	22	Was data saturation discussed?	
Transcripts returned	23	Were transcripts returned to participants for comment and/or	

For peer review only

Topic	Item No.	Guide Questions/Description	Reported on Page No.
		correction?	
**Domain 3: analysis and findings**			
Data analysis			
Number of data coders	24	How many data coders coded the data?	
Description of the coding tree	25	Did authors provide a description of the coding tree?	
Derivation of themes	26	Were themes identified in advance or derived from the data?	
Software	27	What software, if applicable, was used to manage the data?	
Participant checking	28	Did participants provide feedback on the findings?	
Reporting			
Quotations presented	29	Were participant quotations presented to illustrate the themes/findings? Was each quotation identified? e.g. participant number	
Data and findings consistent	30	Was there consistency between the data presented and the findings?	
Clarity of major themes	31	Were major themes clearly presented in the findings?	
Clarity of minor themes	32	Is there a description of diverse cases or discussion of minor themes?	

Developed from: Tong A, Sainsbury P, Craig J. Consolidated criteria for reporting qualitative research (COREQ): a 32-item checklist for interviews and focus groups. *International Journal for Quality in Health Care*. 2007. Volume 19, Number 6: pp. 349 – 357

Once you have completed this checklist, please save a copy and upload it as part of your submission. DO NOT include this checklist as part of the main manuscript document. It must be uploaded as a separate file.

For peer review only

BMJ Open

Exploring how people with dementia can be best supported to manage long-term conditions: A qualitative study of stakeholder perspectives

Journal:	*BMJ Open*
Manuscript ID	bmjopen-2020-041873.R1
Article Type:	Original research
Date Submitted by the Author:	29-Jul-2020
Complete List of Authors:	Rees, Jessica; University College London, Division of Psychiatry<br>Burton, Alexandra; University College London, Division of Psychiatry<br>Walters, Kate; University College London, Primary Care and Population Health<br>Leverton, Monica ; University College London, Division of Psychiatry<br>Rapaport, Penny; University College London, Division of Psychiatry<br>Herat Gunaratne, Ruminda ; University College London, Division of Psychiatry<br>Beresford-Dent, Julie; University of Bradford<br>Cooper, Claudia; University College London
**Primary Subject Heading**:	Qualitative research
Secondary Subject Heading:	Public health
Keywords:	Dementia < NEUROLOGY, PRIMARY CARE, QUALITATIVE RESEARCH

SCHOLARONE™
Manuscripts

I, the Submitting Author has the right to grant and does grant on behalf of all authors of the Work (as defined in the below author licence), an exclusive licence and/or a non-exclusive licence for contributions from authors who are: i) UK Crown employees; ii) where BMJ has agreed a CC-BY licence shall apply, and/or iii) in accordance with the terms applicable for US Federal Government officers or employees acting as part of their official duties; on a worldwide, perpetual, irrevocable, royalty-free basis to BMJ Publishing Group Ltd ("BMJ") its licensees and where the relevant Journal is co-owned by BMJ to the co-owners of the Journal, to publish the Work in this journal and any other BMJ products and to exploit all rights, as set out in our licence.

The Submitting Author accepts and understands that any supply made under these terms is made by BMJ to the Submitting Author unless you are acting as an employee on behalf of your employer or a postgraduate student of an affiliated institution which is paying any applicable article publishing charge ("APC") for Open Access articles. Where the Submitting Author wishes to make the Work available on an Open Access basis (and intends to pay the relevant APC), the terms of reuse of such Open Access shall be governed by a Creative Commons licence – details of these licences and which Creative Commons licence will apply to this Work are set out in our licence referred to above.

Other than as permitted in any relevant BMJ Author's Self Archiving Policies, I confirm this Work has not been accepted for publication elsewhere, is not being considered for publication elsewhere and does not duplicate material already published. I confirm all authors consent to publication of this Work and authorise the granting of this licence.

For peer review only

Exploring how people with dementia can be best supported to manage long-term conditions: A qualitative study of stakeholder perspectives

Authorship:

Jessica Rees[1*], Jessica.rees@ucl.ac.uk

Alexandra Burton[1], a.burton@ucl.ac.uk

Kate Walters[1], k.walters@ucl.ac.uk

Monica Leverton[1], monica.leverton@ucl.ac.uk

Penny Rapaport[1], p.rapaport@ucl.ac.uk

Ruminda Herat-Gunaratne[1], ruminda.hg@gmail.com

Julie Beresford-Dent[2], J.Beresford-Dent@Bradford.ac.uk

and Claudia Cooper[1], claudia.cooper@ucl.ac.uk

[1]University College London, [2]University of Bradford

*Corresponding author: Jessica Rees, UCL Division of Psychiatry, 6th Floor Wing A, Maple House, 149 Tottenham Court Road, London, United Kingdom, W1T 7BN

Key words: Dementia, Long-term Condition, Multi-morbidity, Management, Qualitative, Secondary Analysis, Primary Care.

WORD COUNT: 3990

For peer review only

Abstract

Objectives: To explore how the self-management of comorbid long-term conditions is experienced and negotiated by people with dementia and their carers.

Design: Secondary thematic analysis of 82 semi-structured interviews.

Setting: Community settings across United Kingdom.

Participants: 11 people with dementia, 22 family carers, 19 health professionals and 30 homecare staff.

Results: We identified three overarching themes: 1) *The process of substituting self-management*: Stakeholders balanced the wishes of people with dementia to retain autonomy with the risks of lower adherence to medical treatments. The task of helping a person with dementia to take medication was perceived as intermediate between a personal care and a medical activity; rules about which professionals could perform this activity sometimes caused conflict; 2) *Communication in the care network:* Family carers often communicated with services, and made decisions about how to implement medical advice. In situations where family carers or homecare workers were not substituting self-management, it could be challenging for general practitioners to identify changes in self-management and decide when to intervene. 3) *Impact of physical health on and from dementia*: Healthcare professionals acknowledged the inter-relatedness of physical health and cognition to adapt care accordingly. Some treatments prescribed for long-term conditions were perceived as unhelpful when not adapted to the context of dementia. Healthcare professionals and homecare workers sometimes felt that family carers were unable to accept that available treatments may not be helpful to people with dementia and that this sometimes led to the continuation of treatments of questionable benefit.

Conclusion: The process of substituting self-management evolves with advancement of dementia symptoms and relies on communication in the care network, whilst considering the impact on and from dementia to achieve holistic physical health management. Care decisions must consider people with dementia as a whole, and be based on realistic outcomes and best interests.

Strengths and limitations of the study

- This was a large study involving 82 in-depth interviews with participants.
- Participants were from diverse backgrounds and a range of locations across the UK.
- Interviews included the perspective of homecare staff in addition to people with dementia, family carers and healthcare professionals.
- A limitation to secondary data analysis is that the data were originally collected to explore how to support independence at home for people living with dementia.
- Family carers discussed multi-morbidity most frequently so are relatively over-represented in the analysis.

For peer review only

Introduction

Globally, the prevalence of dementia is predicted to rise to 131.5 million by 2050 compared to 46.8 million in 2015 (1). Over 470,000 people on General Practice registers in England have a formal diagnosis of dementia (2). Almost 8 in every 10 people with dementia live with another chronic long-term disease or condition (3). The health needs within this population are complex (4). The most prevalent multi-morbidities: hypertension, diabetes and coronary heart disease (5), can increase the risk of dementia, while conditions such as stroke, epilepsy and depression are also associated with increased dementia risk (3). The optimal management of physical health may help to prevent hospitalisation and slow cognitive decline (6,7).

Self-management is an integral and lifelong task for people with long-term conditions (8). The symptoms of dementia, including loss of memory, disorientation and executive dysfunction, frequently reduce an individual's ability to organise and plan self-care (9). The most commonly described impact is the diminishing ability to administer medication safely (10,11). Most people with dementia need help to manage care for comorbid long-term conditions – usually from a family carer or homecare worker, or both (12–14). Partnership between this care network at home and primary care is critical for the management of long-term conditions in dementia (15).

Research has highlighted a need for guidance on how to tailor and individualise care for people with dementia and long-term conditions (16,17). Clinical guidelines aim to promote evidence-based best practice but tend to focus on single conditions, so may not reflect difficulties in managing multi-morbidity in dementia (18–20). Understanding the lived experiences of stakeholders is critical to the development of relevant clinical guidelines, including considering interactions between comorbidities on the ability of an individual to self-manage their care (21). The first step towards service development includes understanding the care needs of people living with dementia and co-existing long-term conditions (4). A recent review of the literature found limited evidence about how self-management, and support by other stakeholders, such as homecare workers, can support long-term condition management in dementia (15).

This paper aims to explore the experiences of people living with dementia, family carers, healthcare professionals and homecare staff, to identify how the management of long-term conditions is best supported in dementia.

For peer review only - http://bmjopen.bmj.com/site/about/guidelines.xhtml

Methods

The NIDUS (New Interventions for Independence in Dementia) study (ISRCTN11425138/ ISRCTN99460116) conducted qualitative, semi-structured interviews with people with dementia, family carers, health and social care professionals and homecare staff. The primary analysis of this qualitative data explored how people with dementia can be supported to live as independently as possible in their own homes (22), including a separate analysis on the experiences of South Asian family carers (23). We carried out a secondary analysis of the transcripts to explore the management of long term conditions for people living at home with dementia.

Recruitment

London (Camden and Kings Cross) Research Ethics Services approved the study in November 2017 (reference: 17/LO/1713). People with dementia and family carers were recruited through three UK NHS memory services, private homecare services, an Alzheimer's Society Experts by Experience group and Twitter. Health and social care professionals were recruited through NHS memory services, social services and clinical academics with links to University College London (UCL). For the homecare staff interviews, ten homecare agencies were recruited across England and managers, office support staff and homecare workers were invited to participate. Purposive sampling ensured our sample of people with dementia and family carers was diverse in terms of age, gender, ethnicity, and, for family carers, their relationship to care recipients; and for professionals, their professional roles, and experiences with supporting people with dementia.

Procedure

Participants were invited to take part in semi-structured qualitative interviews between April and September 2018. All participants gave written informed consent. Interviews lasted on average one hour and were conducted in participants' homes, workplaces or at University offices. Interviews followed a semi-structured topic guide which explored how people with dementia live independently at home and what support they need to do so. They were audio-recorded and transcribed verbatim. Across stakeholders, slightly amended versions of the topic guide were used. The interview schedule for people with dementia and family carers included specific questions about how long-term physical or mental health conditions affected the person with dementia's ability to remain living independently at home.

Data analysis

Secondary analysis involves using pre-existing qualitative data collected from previous research studies to investigate new or additional research questions (24). We took an inductive thematic

For peer review only - http://bmjopen.bmj.com/site/about/guidelines.xhtml

approach through focusing on meaning within the data to develop codes (25) using NVivo 12 software (26).

First, the lead author highlighted information in transcripts relating specifically to long-term conditions in addition to dementia. A coding framework was developed with authors (CC, KW, AB) based on line-by-line coding of highlighted sections (see Supplementary Material). The authors systematically labelled codes based on meaningful fragments of transcripts. We met to agree on re-occurring codes, and the lead author applied the coding framework to all transcripts. After completed analysis of all interviews, we reviewed and refined the coding framework by discussing themes corresponding to the research question. We revisited codes looking at commonalities and differences in managing long-term conditions across accounts of all stakeholders for the thematic analysis.

Patient and public involvement

An advisory group of family carers and health professionals provided feedback on the findings from the NIDUS qualitative study which was incorporated into the analysis. We did not directly involve PPI in this secondary analysis, but we have invited members of the advisory group to help us develop our dissemination strategy.

Results

Qualitative interviews were conducted with 11 people with dementia (Mean Age= 78.6, SD=7.8), 22 family carers (Mean age= 57.7, SD= 14.3), 19 health and social care professionals (Mean Age= 41.4, SD= 10.9) and 30 homecare staff (Mean Age= 48.3, SD=11.6). To respond to the needs and preferences of participants, eight interviews were dyadic involving people with dementia and family carers together. Five family carers were born outside the UK. Details of participant characteristics are presented in Tables 1 and 2.

Table 1: Characteristics of participant demographics (person with dementia/family carer)

		Person with dementia	***Family carer***
**Characteristics**		***n***	***n***
Gender	Female	5	12
	Male	6	10
Ethnicity	White British	8	9
	White other	1	0
	Indian	0	6
	Bangladeshi	0	4
	Other	2	3
Living arrangements	Lives alone	5	7
	Lives with relatives	6	15
Type of dementia	Alzheimer’s disease	3	8

For peer review only - http://bmjopen.bmj.com/site/about/guidelines.xhtml

	Vascular	2	4
	Other	2	4
	Not specified	4	6
Relation to person with dementia	Son/daughter	-	11
	Spouse	-	6
	Niece	-	2
	Friend	-	1
	Sibling	-	1
	Daughter in law	-	1

Table 2: Characteristics of participant demographics (professional carers)

		Homecare staff	***Health and social care professional***
**Characteristics**		***n***	***n***
Gender	Female	25	13
	Male	5	6
Ethnicity	White British	22	9
	White other	2	5
	Black/Black British	5	0
	Asian/Asian British	1	0
	Indian	0	2
	Other	0	3
Professional role	Managerial	7	0
	Homecare worker	19	0
	Other Care Staff	4	0
	Commissioner	0	3
	Social worker	0	2
	Dementia lead	0	2
	Service manager	0	2
	Psychologist	0	2
	Support worker	0	2
	GP	0	2
	Geriatrician	0	1
	Nurse	0	1
	Physiotherapist	0	1
	Psychiatrist	0	1

For peer review only

The lead author (JR) extracted information from interview transcripts about long-term conditions discussed across all stakeholders. Interviews with people with dementia (n=9) and family carer (n=22) explicitly spoke about long-term conditions and support needs. Healthcare professionals (n=14) and homecare staff (n=17) included information on specific long-term conditions, but often spoke more generally about medication management and holistic aspects of health for example, falls

For peer review only - http://bmjopen.bmj.com/site/about/guidelines.xhtml

and mobility. Figure 1 presents a full overview of reported long-term conditions by people with dementia, family carers, health and social care professionals and homecare staff.

Figure 1: Frequency of long-term conditions discussed in interviews

[ADD FIGURE 1]

We identified three main themes that responded to our research objectives to explore the experiences of people with dementia, family carers, healthcare professionals and homecare staff in managing long-term conditions in dementia. These were: 1) The process of substituting self-management; 2) Communication in the care network; and 3) Impact on and from dementia (when managing physical health).

1) The process of substituting self-management

Autonomy verses risk

People with dementia valued autonomy when managing their physical health. In the earlier stages of dementia, prompting enabled self-management and a sense of 'still carrying on'. The prospect of requiring more help was a concern to this person living with dementia:

> *"You see I'm praying and I'm hoping that I won't need carers because I want to rely on myself. But I know at some stage I won't be able to. I don't know when. Maybe my health will get worse, physically worse, and this dementia will get worse and I will have to."* (Person with Dementia_1, Arthritis and hypertension)

Homecare workers acknowledged the wishes of people with dementia to retain decision-making control where possible and to be involved in their own care:

> *"I say every time, this is for your memory, this is your thyroid, this is for your Addison's, this is for your blood pressure... And they say, oh thank God you told me because what you're doing is you're controlling them if you don't involve them."* (Homecare Worker_1)

Family carers balanced the wishes of people living with dementia to retain autonomy as far as possible with the potential consequences of lower adherence to medical treatment. Family carers described their discomfort in providing care against the wishes of the person with dementia but acknowledged the importance of such tasks when memory impeded medication administration:

> *"He then lost his eyesight in the left eye, due to the diabetes medication being missed. He was reporting to my mother daily that he is consuming his medication. Of course, because of the dementia, his memory was deceiving him and telling him that he had done it, but this was a past memory"* (Family Carer_1, Son)

For peer review only - http://bmjopen.bmj.com/site/about/guidelines.xhtml

Prescriptions and role restrictions

Monitoring or administering medication was the most frequently discussed form of support for long-term condition management. While self-administration of medication is a routine part of long-term condition management, when a person with dementia required support of another person, this role occupied an intermediate position between a personal care activity, and a medical task that required a nursing qualification. Rules around who administered medication could be complex and confusing:

> *"Although we were told that the [home]carers are not allowed to medicate, because they're not insured. So, that's a difficult one."* (Family Carer_2, Son)

One healthcare professional described the difficulties that could arise because of this when prescriptions changed:

> *"It falls through when they're on, say steroids, like emergency steroids if they've got COPD or if they're on antibiotics for infection. Where the person with dementia will get really poorly because they're in boxes and [home]carers can't give out boxes; it has to be in a nomad [pre-packed medication]. ."* (Community mental health nurse)

2) Communication in the care network

In healthcare settings, it was often the family carer who communicated with services, and made decisions about how to implement medical advice. Being an implementer of medical advice but not the doctor, and the recipient of advice but not the patient, sometimes felt like a dilemma for family carers working as in partnership with people with dementia:

> *"…. I suppose at the end of the day I should go down and make an appointment to see the GP…. to tell them that I feel she needs that done…. but I don't want to go down and sort of feel that I'm…. telling them what to do, coming heavy-handed on it either.. "* (Family Carer_3, Sister)

Family carers experienced additional challenges in communication when developed relationships with primary care came to an end, as changes in practitioner's impacted continuity of care.

Where people with dementia experienced difficulties with communication, family carers offered support in healthcare appointments to ensure correct reporting of symptoms.

> *"…she would tell them things that were factually incorrect and they would believe that they were correct. Because they just thought she might be a bit old and her literacy wasn't great…But she was on drugs that were negatively affecting her …she was on something that were actually negative to her heart."* (Family Carer_4, Son)

For peer review only - http://bmjopen.bmj.com/site/about/guidelines.xhtml

Regardless of the presence of a proxy, participants with dementia highlighted the importance of continued acknowledgement within appointments:

> *"It was, I think that's the problem, some people, because you've got diagnosed with it they think, oh he can't digest this, we'll talk to the relative"* (Person with Dementia_2, Visual Impairment)

In situations where family carers or homecare workers were not substituting self-management, it could be challenging for general practitioners to identify changes in self-management and decide when to intervene:

> *"But I'm not quite sure that the mechanisms are very good for picking up on that, you know, the bit where they're in between, where they're potentially sort of just well enough to go out and about, and do things for themselves, but maybe things at home aren't good and they're not coping, and actually, as a GP, maybe it's really difficult to pick up on that."* (General Practitioner_1)

3) Impact on and from dementia (when managing physical health)

Inter-relatedness of cognition and physical health

As the outcomes associated with physical health and cognition were found to be inter-related, healthcare professionals sought to optimise physical and preventative healthcare in ways that improved cognition.

> *"Yes, so, I think, you know, the physical health of someone has a really big impact on how they're dementia... they experience their dementia. So, they might just have a really poor diet which means that... or they might not be able to manage their diabetes and therefore they're feeling rubbish all the time."* (Memory Service Manager)

> *"Physical problems as well. You know? Because they have, not only possible dementia, but they also may have physical illnesses. Parkinson's, stroke, any sort of medical condition, as well, on top. So, we don't just concentrate on the dementia. We concentrate on the person. And it's a very much a holistic approach."* (Occupational therapist)

Limitations of physical health treatments in people with dementia

Some treatments prescribed for long-term conditions were considered to be unhelpful for people with dementia, for example because the strategies they used depended on memory, and could not or had not been adapted to the context of dementia. In this next quote, a family carer explains how a person with dementia was too impaired to benefit from suggested strategies:

For peer review only - http://bmjopen.bmj.com/site/about/guidelines.xhtml

"Well, she did have a visit from an occupational therapist [following a stroke]. They arranged that for a few weeks but he, it was when she was at her most florid time and he was recommending that she did things like writing a list down of things that interested her and what she would like to do and things like that. But she wouldn't do any of those. And he said, you know, he came about four or five times, no point in me coming because she doesn't take any notice of what I say, or do anything." (Family Carer_3, Sister)

Healthcare professionals and homecare workers sometimes felt that family carers were unable to accept that available treatments may not be helpful to people with dementia. They reported that this sometimes led to the continuation of treatments of questionable benefit, even when it went against the preferences of the person with dementia receiving them:

"I suppose family members are sometimes trying to keep people as they would see them, well and physically well for as long as possible and doing these things that their diabetes or anything else, and so they'll want them to do certain things whereas that's no longer what is important to that person." (General Practitioner_2)

"I have a lady that's quite old and her mother, she wants the physio in and it's just not possible. She physically can't do it, but she thinks it'll keep her strong a little bit. And it's really not doing her any good, even the physio said that. But the daughter wants it." (Homecare Worker_2)

The above quotes highlight how the voice of people living with dementia can be lost when determining the benefits of treatment. Family carers appear to adopt an 'all or nothing' approach to physical health decision-making due to the lack of flexible interventions which successfully account for dementia.

At other times, family carers and professionals discussed and agreed approaches in collaboration with people with dementia. One family carer described how this was helpful where difficult, end of life decisions, balancing comfort with quality of life needed to be taken:

"Kidney is only about 6% working. So because of that, you know... And we've decided not to go on dialysis. Not just we, but the specialist kidney doctor, we went to see him a couple of months ago and he suggested as well not to put him on dialysis because of his age…And he didn't think it was going to be beneficial to him…." (Family Carer_5, Daughter)

For peer review only - http://bmjopen.bmj.com/site/about/guidelines.xhtml

Discussion

Main findings

We describe three themes exploring how the self-management of comorbid long-term conditions is experienced and negotiated by people with dementia and their carers.

The first theme focused on the transition of roles which occurred in relation to advancement of dementia symptoms, with support increasing in accordance to level of need. Substituting self-management, especially medication, presented conflicts for family carers and homecare staff, to balance involving people with dementia in their care with safety concerns. The second theme identified communication as a key concept in the management of long-term conditions in dementia. Partnership working between people with dementia and family carers sought to overcome communication difficulties as a result of dementia. Without a family carer, changes in an individual's ability to self-manage may be difficult to detect, especially when people with dementia infrequently accessed primary care. The final theme highlighted the inter-relatedness of cognition and physical health and the importance of adapting physical healthcare to the context of dementia. Best interest decision making is likely to be improved with increased awareness of likely harms and benefits of treatment based on evidence such as age and stage of dementia.

Addressing the research gap recently identified in the field of dementia and multi-morbidity, these findings describe the interacting effects between cognition and physical health and provide an understanding of the care needs of this population (4,21). In dementia care, family carers often experienced a role transition from a 'care-partner' (who provides assistance to a person who continues to manage much of their own care themselves) to 'care-giver' (who provides care) with increasing disability (12). Our analysis demonstrates the critical role of family carers in communicating with primary care (13). In addition to impacting memory, judgment and orientation, the impact of dementia on language skills has previously been identified as a barrier to reporting symptoms in healthcare appointments (9). Relevant theoretical models of independence at home in dementia suggest that professionals should also be viewed as partners, rather than experts, when supporting self-management (27). The integrated logic of care model suggests that psychosocial and physical needs have a great influence on each other, and should therefore be addressed simultaneously (28). Our analysis highlights a similar need for physical health and cognition.

One of the main self-management tasks across long-term conditions is medication adherence (8,9). Similar to the findings of Rapaport et al (2020), our analysis highlights the role of homecare workers in adapting a facilitative approach; specifically 'doing with not for' during medication management. The need for both family carers and homecare workers to prevent harm often conflicts with the

For peer review only - http://bmjopen.bmj.com/site/about/guidelines.xhtml

desire of a person with dementia to remain autonomous (29). The balancing of roles is complex for carers especially involving boundaries between advocating for independence and the implementation of medical advice (30). Our findings demonstrate this dilemma extends to the management of long-term conditions in dementia. Future research is required to understand how calculated risks in care decision making can enable freedom for people with dementia whilst managing carer safety concerns (27). Living well with dementia has been conceptualised as living with quality of life, choice, autonomy, dignity and as independently as possible. There can be a tension between independence as an expression of autonomy and interdependence that can enable people with dementia to live in their own homes for longer People with dementia and carers often have different goals and priorities for care. Judging at which point a person loses capacity to refuse care, and when to intervene in their best interests can be challenging. Research has indicated the need for professionals to facilitate conversations around the decision to move to care homes (31). A similar approach may be required during physical health decision making.

One of the five core self-management skills is decision-making (8) impacted by dementia due to impairments in executive functioning (9). Non-dementia health decisions, such as end of life care, has previously been identified as a problematic decision-making area for family carers (32). In this context, care decisions involving all stakeholders require a focus on quality of life over a target-driven approach (16) which is continuously re-assessed according to changes in disease state (17). Quality of life is an important indicator of effectiveness for person-centred care interventions in dementia (33).

Clinical implications

These findings highlight that management of long-term conditions in dementia exists on a continuum with support from all stakeholders developing across stages of dementia and severity of long-term conditions. To best support people with dementia to manage long-term conditions, treatments should be congruent to need and consider the impact of dementia on engagement. The development of dementia specific consultation models must consider the impact of dementia on language skills and communication ability, and work with family carers acting in dyadic care relationships with people with dementia. Greater considerations of how healthcare professionals communicate the limitations of treatments that are not in the best interest or of questionable benefit to people with dementia and family carers would improve physical health decision-making. Such discussions are often complex, involving various stakeholders and ensuring the voice of people with dementia is heard when deciding on treatment benefits can be challenging. Service development must identify mechanisms for detecting change in self-management ability when

For peer review only - http://bmjopen.bmj.com/site/about/guidelines.xhtml

family carer or homecare workers are not involved in supporting self-management at home for example, alerts for appointment non-attendance and medication under-utilisation.

Strengths and limitations

To our knowledge, this paper presents the first qualitative analysis to consider how people with dementia can be best supported to manage long-term conditions from the perspective of all stakeholders. Specifically, this paper expands on previous research by considering the role of the homecare worker in the care network, previously described in the literature as a triad between the person with dementia, their family carers, and healthcare professionals (34,35). The analysis includes interviews from a large and varied sample from a range of locations across the UK.

We used secondary analysis to explore sections of transcripts that discuss multi-morbidity in dementia. A limitation of this analysis approach is that the data was originally collected to explore how to support independence at home in dementia. Nevertheless, participants spontaneously discussed experiences of long-term conditions due to their salience, enabling further exploration of multi-morbidity in this analysis. All family carers explicitly discussed the management of long-term conditions which may account for large representation from this stakeholder in the analysis, while people with dementia, healthcare professionals and homecare workers would discuss more generally about medication management and physical health.

We included co-authors involved in original data collection (AB, ML, JBD, RHG) to bring an inside knowledge of context during analysis to overcome the problem of the lead author 'not having been there' (24). In addition, the lead author listened to recordings of transcripts to become familiar with data. The use of additional methods such as participant observations in future research would enable triangulation of results on how stakeholders can best support people with dementia to manage long-term conditions (36).

We purposively recruited a diverse sample in terms of ethnicity and gender, and while we considered during analysis how these factors might intersect our themes, unlike in our previous publications from this database on different topics (22,23), we did not find any clear areas where themes were gendered or were reflected differently across ethnic groups or immigration status (37). While this was part of the reflective analytic process, we did not include an intersectionality analysis as an a priori analytic step. Williams and Colleagues did this, and we will consider adapting this approach in our future work (38).

For peer review only - http://bmjopen.bmj.com/site/about/guidelines.xhtml

Conclusion

Family carers and homecare workers support the management of long-term conditions in the home. Family carers act as proxies for communication in healthcare appointments. Healthcare professionals have a responsibility to identify changes in self-management ability as dementia progresses; and adapt care accordingly. This process of substituting self-management evolves with advancement of dementia symptoms and relies on communication in the care network, whilst considering the impact on and from dementia to achieve holistic physical health management. Care decisions must consider people with dementia as a whole, and be based on realistic outcomes and best interests.

Declarations

Declaration of interests

The authors declare that they have no known competing financial interests or personal relationships that could have appeared to influence the work reported in this paper.

Funding statement

This work was supported by the Economic and Social Research Council [grant number ES/P0 0 0592/1]; and the National Institute of Health Research (NIHR) Collaboration for Leadership and Applied Health Research (North Thames). The views expressed are those of the author(s) and not necessarily those of the NIHR or the Department of Health and Social Care.

Data collection was carried out within the UCL Alzheimer's Society Centre of Excellence for Independence at home, NIDUS (New Interventions in Dementia Study) programme (Alzheimer's Society Centre of Excellence grant 330).

Author contribution

All authors made a substantial contribution to this work. JR, CC, KW and AB all contributed to the conception and design of the study and JR drafted the paper. All authors critically revised it and gave final approval for this version to be published. AB, ML, JBD, PR and RHG collected the qualitative data. JR, CC, KW, and AB all coded some of the interview transcripts. JR then organised the data into preliminary themes and led the subsequent analysis, CC, KW and AB further refined the thematic analysis in an iterative process.

Data sharing statement

The qualitative data used and analysed during the current study are available from the corresponding author on reasonable request.

For peer review only

References

1. Prince M, Wimo A, Guerchet M, Ali GC, Wu YT, Prina M. World Alzheimer Report 2015. London, UK. Alzheimer's Dis Int [Internet]. 2015;1–92. Available from: https://www.alz.co.uk/research/WorldAlzheimerReport2015.pdf

2. NHS Digital. Recorded Dementia Diagnoses - March 2020 [Internet]. 2020 [cited 2020 May 1]. Available from: https://digital.nhs.uk/data-and-information/publications/statistical/recorded-dementia-diagnoses/march-2020

3. Public Health England. Dementia: comorbidities in patients - GOV.UK [Internet]. 2019 [cited 2020 Apr 27]. Available from: https://www.gov.uk/government/publications/dementia-comorbidities-in-patients

4. Welsh. Multimorbidity in people living with dementia. Case Reports Women's Heal. 2019;23:e00125.

5. Browne J, Edwards DA, Rhodes KM, Brimicombe DJ, Payne RA. Association of comorbidity and health service usage among patients with dementia in the UK: A population-based study. BMJ Open. 2017;7(3):1–8.

6. Bordier L, Doucet J, Boudet J, Bauduceau B. Update on cognitive decline and dementia in elderly patients with diabetes. Diabetes Metab [Internet]. 2014 Nov 1 [cited 2019 Sep 20];40(5):331–7. Available from: https://www.sciencedirect.com/science/article/pii/S1262363614000366?via%3Dihub

7. Doraiswamy PM, Leon J, Cummings JL, Marin D, Neumann PJ. Prevalence and Impact of Medical Comorbidity in Alzheimer's Disease. Journals Gerontol Ser A Biol Sci Med Sci [Internet]. 2002 Mar 1 [cited 2019 Aug 23];57(3):M173–7. Available from: https://academic.oup.com/biomedgerontology/article-lookup/doi/10.1093/gerona/57.3.M173

8. Lorig KR, Holman HR. Self-management education: History, definition, outcomes, and mechanisms. Ann Behav Med [Internet]. 2003 Aug [cited 2019 Jun 10];26(1):1–7. Available from: http://www.ncbi.nlm.nih.gov/pubmed/12867348

9. Ibrahim J., Anderson. L., Davis., M C, MacPhail. A, Lovell. J, Winbolt. M. Chronic disease self-management support for persons with dementia, in a clinical setting. J Multidiscip Healthc. 2017;10:49–58.

10. Elliott RA, Goeman D, Beanland C, Koch S. Ability of Older People with Dementia or Cognitive Impairment to Manage Medicine Regimens: A Narrative Review. Curr Clin Pharmacol [Internet]. 2015 [cited 2019 Aug 23];10:213–21. Available from: https://www.ncbi.nlm.nih.gov/pmc/articles/PMC5396255/pdf/CCP-10-213.pdf

11. Kaasalainen S, Dolovich L, Papaioannou A, Holbrook A, Lau E, Ploeg J, et al. The process of medication management for older adults with dementia. J Nurs Healthc Chronic Illn [Internet]. 2011 Dec 1 [cited 2019 Aug 23];3(4):407–18. Available from: http://doi.wiley.com/10.1111/j.1752-9824.2011.01114.x

12. Lazaroff A, Morishita L, Schoephoerster G, McCarthy T. Using dementia as the organizing principle when caring for patients with dementia and comorbidities. Minn Med. 2013 Jan;96(1):41–6.

13. Health Education England. Managing success in dementia care [Internet]. 2018. Available from: https://www.hee.nhs.uk/sites/default/files/documents/Managing Success v3.pdf

For peer review only - http://bmjopen.bmj.com/site/about/guidelines.xhtml

14. Allen S. Better domiciliary care for people with dementia Preface [Internet]. 2014 [cited 2020 Apr 27]. Available from: www.skillsforcare.org.uk

15. Rees J, Tuijt R, Burton A, Walters K, Cooper C. Supporting self-care of long-term conditions in dementia: A systematic review. Int J Nurs Stud. 2020 Sep;103432.

16. Bunn F, Goodman C, Jones PR, Russell B, Trivedi D, Sinclair A, et al. Managing diabetes in people with dementia: A realist review. Health Technol Assess (Rockv). 2017;21(75):1–139.

17. Baird C, Woolford MH, Young C, Winbolt M, Ibrahim J. Chronic disease management and dementia: a qualitative study of knowledge and needs of staff. Aust J Prim Health. 2019;25(4):359–65.

18. Guthrie B, Payne K, Alderson P, McMurdo MET, Mercer SW. Adapting clinical guidelines to take account of multimorbidity. BMJ. 2012;345(7878):1–5.

19. Mutasingwa DR, Ge H, Upshur REG. How applicable are clinical practice guidelines to elderly patients with comorbidities? Vol. 57, Canadian Family Physician. 2011.

20. NICE. Multimorbidity: clinical assessment and management. 2016;

21. Subramaniam H. Co-morbidities in dementia: Time to focus more on assessing and managing co-morbidities. Age Ageing. 2019;48(3):314–5.

22. Rapaport P, Burton A, Leverton M, Herat-Gunaratne R, Beresford-Dent J, Lord K, et al. "I just keep thinking that I don't want to rely on people." a qualitative study of how people living with dementia achieve and maintain independence at home: stakeholder perspectives. BMC Geriatr. 2020 Jan 3;20(1):5.

23. Herat-Gunaratne R, Cooper C, Mukadam N, Rapaport P, Leverton M, Higgs P, et al. "in the Bengali Vocabulary, There Is No Such Word as Care Home": Caring Experiences of UK Bangladeshi and Indian Family Carers of People Living with Dementia at Home. Gerontologist [Internet]. 2020 [cited 2020 Mar 5];60(2):331–9. Available from: https://academic.oup.com/gerontologist/article-abstract/60/2/331/5581953

24. Heaton J. Secondary analysis of qualitative data: an overview. Hist Soc Res. 2008;33(3):33–45.

25. Braun V, Clarke V. Using thematic analysis in psychology. Qual Res Psychol [Internet]. 2006 [cited 2019 Aug 28];3(2):77–101. Available from: https://www.tandfonline.com/action/journalInformation?journalCode=uqrp20

26. QSR International Pty. NVivo qualitative data analysis software. 2018. p. Version 12.

27. Lord K, Beresford-Dent J, Rapaport P, Burton A, Leverton M, Walters K, et al. Developing the New Interventions for independence in Dementia Study (NIDUS) theoretical model for supporting people to live well with dementia at home for longer: a systematic review of theoretical models and Randomised Controlled Trial evidence [Internet]. Vol. 55, Social Psychiatry and Psychiatric Epidemiology. Springer; 2020 [cited 2020 Jul 27]. p. 1–14. Available from: https://doi.org/10.1007/s00127-019-01784-w

28. Hansen A, Hauge S, Bergland Å. Meeting psychosocial needs for persons with dementia in home care services - A qualitative study of different perceptions and practices among health care providers. BMC Geriatr. 2017;17(1):1–10.

29. Smebye KL, Kirkevold M, Engedal K. Ethical dilemmas concerning autonomy when persons with dementia wish to live at home: A qualitative, hermeneutic study Healthcare needs and demand. BMC Health Serv Res [Internet]. 2016 Jan 19 [cited 2020 Apr 28];16(1):21. Available from: http://bmchealthservres.biomedcentral.com/articles/10.1186/s12913-015-1217-1

For peer review only - http://bmjopen.bmj.com/site/about/guidelines.xhtml

30. Leverton M, Burton A, Rees J, Rapaport P, Manthorpe J, Downs M, et al. A systematic review of observational studies of adult home care. Health Soc Care Community [Internet]. 2019 Nov 22 [cited 2019 Oct 17];27(6):1388–400. Available from: https://onlinelibrary.wiley.com/doi/abs/10.1111/hsc.12831

31. Lord K, Livingston G, Robertson S, Cooper C. How people with dementia and their families decide about moving to a care home and support their needs: development of a decision aid, a qualitative study. BMC Geriatr [Internet]. 2016 Dec 21 [cited 2019 Sep 23];16(1):68. Available from: http://www.biomedcentral.com/1471-2318/16/68

32. Livingston G, Leavey G, Manela M, Livingston D, Rait G, Sampson E, et al. Making decisions for people with dementia who lack capacity: Qualitative study of family carers in UK. BMJ [Internet]. 2010 Sep 4 [cited 2020 Jul 27];341(7771):494. Available from: http://www.bmj.com/

33. Kim SK, Park M. Effectiveness of person-centered care on people with dementia: A systematic review and meta-analysis [Internet]. Vol. 12, Clinical Interventions in Aging. Dove Medical Press Ltd.; 2017 [cited 2020 Jul 27]. p. 381–97. Available from: /pmc/articles/PMC5322939/?report=abstract

34. Fortinsky RH. Health care triads and dementia care: Integrative framework and future directions. In: Aging and Mental Health. 2001.

35. Bunn F, Burn A-M, Goodman C, Robinson L, Rait G, Norton S, et al. Comorbidity and dementia: a mixed-method study on improving health care for people with dementia (CoDem). Heal Serv Deliv Res. 2016;4(8):1–156.

36. Mays N, Pope C. Qualitative research in health care Assessing quality in qualitative research [Internet]. Vol. 320, BMJ VOLUME. 2000 [cited 2020 Apr 28]. Available from: www.bmj.com

37. Rote SM, Moon H, Kent RA, Rote S. Racial/Ethnic Differences in Caregiving Frequency: Does Immigrant Status Matter? [cited 2020 Jul 28]; Available from: https://academic.oup.com/psychsocgerontology/article-abstract/73/6/1088/2631947

38. Williams A, Sethi B, Duggleby W, Ploeg J, Markle-Reid M, Peacock S, et al. A Canadian qualitative study exploring the diversity of the experience of family caregivers of older adults with multiple chronic conditions using a social location perspective [Internet]. Vol. 15, International Journal for Equity in Health. BioMed Central Ltd.; 2016 [cited 2020 Jul 28]. p. 40. Available from: http://www.equityhealthj.com/content/15/1/40

For peer review only

For peer review only - http://bmjopen.bmj.com/site/about/guidelines.xhtml

Figure 1: Frequency of long-term conditions discussed in interviews

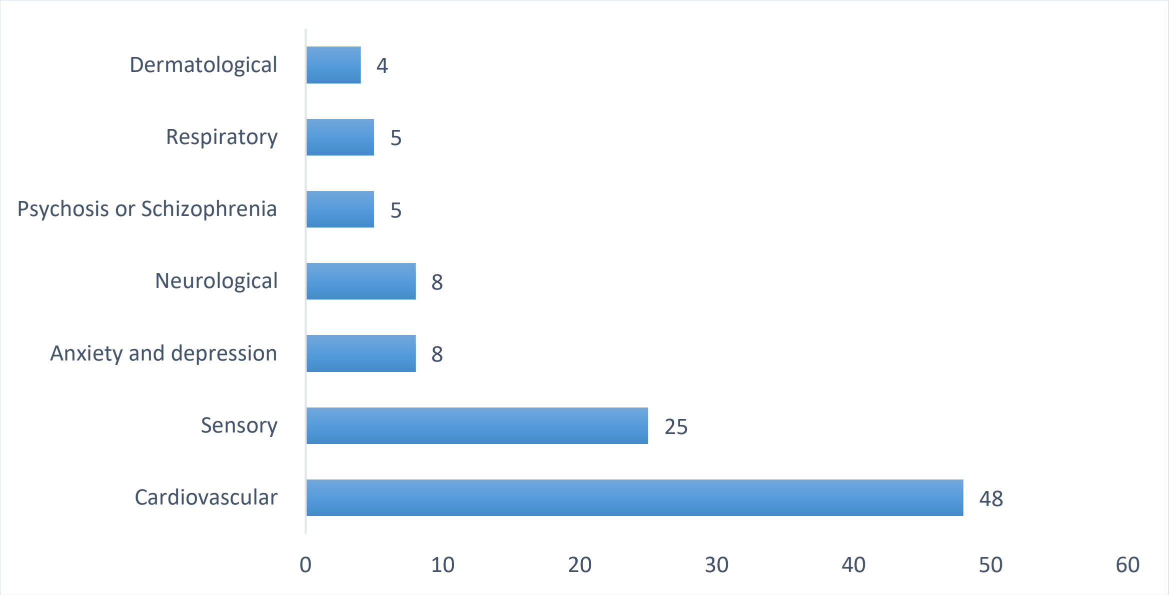

NB:

Category	Long-term condition
**Mental health**	Anxiety and depression<br>Psychosis and Schizophrenia
**Sensory**	Visual impairment<br>Hearing impairment
**Musculoskeletal**	Arthritis<br>Osteoporosis
**Respiratory**	Asthma<br>Pulmonary fibrosis<br>Chronic obstructive pulmonary disease
**Cardiovascular**	Diabetes<br>Hypertension<br>Heart problems<br>Stroke
**Dermatological**	Eczema<br>Psoriasis<br>Dermatitis

For peer review only

PROCESS OF SUBSTITUTING SELF-MANAGEMENT	Files	References
**Autonomy verses risk**	0	0
Consequences of non-adherence	2	3
Independence	5	8
Lock safe for medication	4	7
Reliance on others	3	6
Risk management	9	11
Safety concerns	8	14
**Stages of dementia**	5	5
Ability to self-manage	9	9
Capacity	5	8
Managing a spectrum of needs	5	6
Support increasing with level of need	7	9
Taking responsibility for tasks	8	11

For peer review only - http://bmjopen.bmj.com/site/about/guidelines.xhtml

**Support in medication management**	0	0
Blister packs	12	18
Encouragement to take medication	5	5
Family member managing medication	15	20
Homecarer role in medication management	26	63
Training to improve limits to HCW role	6	9
Memory aids	8	9
Prompts	9	11
Role of HCP in medications	7	8
Understanding medication	13	15

For peer review only - http://bmjopen.bmj.com/site/about/guidelines.xhtml

COMMUNICATION IN THE CARE NETWORK	Files	References
**Appointment**	13	25
Family carer support in medical appointments	14	23
Organising support and care	12	14
Seeking and providing advice	3	4
**Continuity of care**	8	12
Relationships	7	8
Understanding PLWD	8	9
**Monitoring change**	11	12
Medication review	8	9
Review and monitoring	8	8
**PLWD communication**	9	12
Communication difficulties	7	12
Family carers proxy	4	4

For peer review only - http://bmjopen.bmj.com/site/about/guidelines.xhtml

IMPACT ON AND FROM DEMENTIA	Files	References
Aligned goals and different priorities	5	9
**Decision making**	8	9
**Inter-relateness of physical health and cognition**	0	0
Dementia	7	8
Link between cognition and physical health	15	29
Managing dementia when supporting physical health	7	11
Physical health problems resulting in dementia diagnosis	8	12
**Services congruent to need**	1	1
Accessibility	17	27
Dementia services	3	5
HCP frustration with use of services	1	4

For peer review only - http://bmjopen.bmj.com/site/about/guidelines.xhtml

COREQ (COnsolidated criteria for REporting Qualitative research) Checklist

A checklist of items that should be included in reports of qualitative research. You must report the page number in your manuscript where you consider each of the items listed in this checklist. If you have not included this information, either revise your manuscript accordingly before submitting or note N/A.

Topic	Item No.	Guide Questions/Description	Reported on Page No.
**Domain 1: Research team and reflexivity**			
Personal characteristics			
Interviewer/facilitator	1	Which author/s conducted the interview or focus group?	
Credentials	2	What were the researcher's credentials? E.g. PhD, MD	
Occupation	3	What was their occupation at the time of the study?	
Gender	4	Was the researcher male or female?	
Experience and training	5	What experience or training did the researcher have?	
Relationship with participants			
Relationship established	6	Was a relationship established prior to study commencement?	
Participant knowledge of the interviewer	7	What did the participants know about the researcher? e.g. personal goals, reasons for doing the research	
Interviewer characteristics	8	What characteristics were reported about the inter viewer/facilitator? e.g. Bias, assumptions, reasons and interests in the research topic	
**Domain 2: Study design**			
Theoretical framework			
Methodological orientation and Theory	9	What methodological orientation was stated to underpin the study? e.g. grounded theory, discourse analysis, ethnography, phenomenology, content analysis	
Participant selection			
Sampling	10	How were participants selected? e.g. purposive, convenience, consecutive, snowball	
Method of approach	11	How were participants approached? e.g. face-to-face, telephone, mail, email	
Sample size	12	How many participants were in the study?	
Non-participation	13	How many people refused to participate or dropped out? Reasons?	
Setting			
Setting of data collection	14	Where was the data collected? e.g. home, clinic, workplace	
Presence of non-participants	15	Was anyone else present besides the participants and researchers?	
Description of sample	16	What are the important characteristics of the sample? e.g. demographic data, date	
Data collection			
Interview guide	17	Were questions, prompts, guides provided by the authors? Was it pilot tested?	
Repeat interviews	18	Were repeat inter views carried out? If yes, how many?	
Audio/visual recording	19	Did the research use audio or visual recording to collect the data?	
Field notes	20	Were field notes made during and/or after the inter view or focus group?	
Duration	21	What was the duration of the inter views or focus group?	
Data saturation	22	Was data saturation discussed?	
Transcripts returned	23	Were transcripts returned to participants for comment and/or	

For peer review only

Topic	Item No.	Guide Questions/Description	Reported on Page No.
		correction?	
**Domain 3: analysis and findings**			
Data analysis			
Number of data coders	24	How many data coders coded the data?	
Description of the coding tree	25	Did authors provide a description of the coding tree?	
Derivation of themes	26	Were themes identified in advance or derived from the data?	
Software	27	What software, if applicable, was used to manage the data?	
Participant checking	28	Did participants provide feedback on the findings?	
Reporting			
Quotations presented	29	Were participant quotations presented to illustrate the themes/findings? Was each quotation identified? e.g. participant number	
Data and findings consistent	30	Was there consistency between the data presented and the findings?	
Clarity of major themes	31	Were major themes clearly presented in the findings?	
Clarity of minor themes	32	Is there a description of diverse cases or discussion of minor themes?	

Developed from: Tong A, Sainsbury P, Craig J. Consolidated criteria for reporting qualitative research (COREQ): a 32-item checklist for interviews and focus groups. *International Journal for Quality in Health Care*. 2007. Volume 19, Number 6: pp. 349 – 357

Once you have completed this checklist, please save a copy and upload it as part of your submission. DO NOT include this checklist as part of the main manuscript document. It must be uploaded as a separate file.

For peer review only
